# Modified lumbar foraminoplasty using a power-aided reciprocating burr for percutaneous transforaminal endoscopic lumbar discectomy: A technical note and clinical report

**DOI:** 10.3389/fsurg.2022.1091187

**Published:** 2023-01-05

**Authors:** Yingbo Wang, Jian Wu, Tengyu Wang, Yaoyao Liu, Mei Jiang, Zhong Wang, Rui Chao, Peng Liu, Jungang Pu, Weili Fan

**Affiliations:** ^1^ Department of Spine Surgery, Center for Orthopedics, Daping Hospital, Army Medical University, Chongqing, China; ^2^ Department of Orthopedics, The Third Affiliated Hospital of Chongqing Medical University, Chongqing, China; ^3^ Department of Orthopedics, Chongqing Emergency Medical Center, Chongqing University, Chongqing, China

**Keywords:** foraminoplasty, disc degeneration, lumbar discectomy, endoscope, learning curve

## Abstract

**Background:**

One of the main difficulties in a transforaminal endoscopic lumbar discectomy (TELD), and simultaneously the most critical step, is performing an effective and safe foraminoplasty, which is especially difficult for beginners. To make it safer and faster for beginners to perform, we have used a specially designed power-aided reciprocating burr for TELD and reported the technical details.

**Methods:**

From Jan. 2019 to Nov. 2022, 432 patients with single-level, symptomatic L4/5 or L5/S1 disc herniation were treated with TELD using a novel power-aided reciprocating burr. The surgical procedure is described in detail. Magnetic resonance imaging (MRI) was performed the following day and 3 months after the operation. The learning curves of surgeons with different seniority levels are displayed. The Visual Analogue Scale (VAS) score and the Oswestry Disability Index (ODI) were used to measure low back pain, leg pain, and lumbar function. All patients were followed up for at least 1 year.

**Results:**

All patients underwent endoscopic surgery successfully. Among the 432 patients, radicular outer membrane damage was observed in 6 cases, and 1 case had hernia of the nerve tract. Except for this patient with aggravation of postoperative numbness, the postoperative neurological symptoms of all patients were significantly improved. The mean VAS scores for low back pain and leg pain and ODI scores were significantly decreased 6 w post-operatively and were maintained until 12 months post-operatively compared to preoperative scores (*P* < 0.05). All three doctors involved in the study had substantial experience in traditional open spinal surgery. The more operations all three surgeons completed, the more time spent on intervertebral foraminoplasty decreased (*P* < 0.05). Among them, doctors without experience in TELD surgery became proficient in this technique after accumulating experience in 13 cases. There was no significant difference in foraminoplasty time among these three surgeons during the same growing period (*P* > 0.05).

**Conclusions:**

Current clinical data demonstrated the safety and efficacy of modified TELD using a power-aided reciprocating burr for treating lumbar disc herniation (LDH) and showed that this technique significantly reduces the learning curve for beginners when performing foraminoplasty.

## Introduction

Lumbar disc herniation (LDH) is one of the most common spinal degenerative disorders that can cause low back pain (LBP) and radicular leg pain. Patients who do not benefit significantly from strict conservative treatment should consider surgery. Percutaneous transforaminal endoscopic lumbar discectomy (TELD) is a minimally invasive surgical procedure performed while the patient is awake through an incision of no more than 1 cm in length. Compared with traditional open surgery, TELD is preferred due to the advantages of less pain, less paravertebral muscle injury, preservation of the posterior ligamentous, and faster recovery (1–3). Despite the remarkable evolution of endoscopic techniques and instrumentation, traditional TELD requires extensive training for surgeons to overcome its steep learning curve (4–6). One of the main difficulties in TELD, and simultaneously the most critical step, is performing an effective and safe foraminoplasty.

Foraminoplasty is the enlargement of the foramen by cutting the superior articular process (SAP) end with bone trephines, side-firing laser, reamers, endoscopic round diamond burr, etc (7–10). Advances in endoscopic equipment, such as endoscopic burrs through the endoscope's working channel, have improved the optical system and provided the foundation for developing other endoscopic surgical techniques (11, 12). A fully endoscopic burr or trephine may further improve the safety of foraminoplasty to some extent. However, endoscopic foraminoplasty with tiny tools and a burr is a time-consuming procedure because of the size restriction of the working channel of the rigid endoscope. At the same time, the surgeon must be familiar with the anatomy of the foraminal region. In addition, the increase in temperature while using a high-speed burr may lead to inflammation of the nerve and may cause deterioration of nerve conduction to some extent (13). The trephine can quickly cut off the hypertrophied SAP or osteophyte under fluoroscopic guidance. It is more efficient and time saving than endoscopic foraminoplasty (14). Nevertheless, even with a protective working cannula, it carries the risk of injury to the exiting and traversing nerve root, which may produce leg pain and neurological dysfunction in the affected extremity. The trephine has other disadvantages, such as serrations that are too sharp, more radiation, and a steep learning curve (15, 16, 17). Therefore, lumbar foraminoplasty, especially for beginners, is still challenging.

To make it safer and faster for beginners to perform, we used a specially designed power-aided reciprocating burr for percutaneous lumbar foraminoplasty. The purpose of this study was to present a modified lumbar foraminoplasty using a specially designed burr and report the technical details and clinical outcomes.

## Material and methods

### Participants

From Jan. 2019 to Nov. 2021, 432 patients with lumbar disc herniation (LDH) were included in this retrospective study, including 257 males and 175 females, with an average age of 49.0 (19–75) years. These patients were diagnosed with single-level lumbar disc herniation according to symptoms, signs, and MRI results (L4/5 in 230 cases and L5/S1 in 202 cases). The study was approved by the ethics committee of the Army Medical Center of PLA (IRB approval number: 2018117) and was conducted according to the principles of the Declaration of Helsinki. It was registered in the Chinese Clinical Trial Registry (ChiCTR1900028671).

The inclusion criteria were as follows: (1) single-level lumbar disc herniation and unilateral radicular leg pain, (2) conservative and ineffective treatment for 6–8 weeks, (3) MRI showing symptoms and signs consistent with the respective segment, and (4) willingness to undergo endoscopic surgery. The following exclusion criteria were used: (1) segmental instability on preoperative extension/flexion radiographs, (2) severe central stenosis on preoperative MRI or CT, (3) L5/S1 LDHs with an iliac crest higher than the L4/5 disc level, (4) other diseases and the inability of the patient to tolerate surgery, and (5) recurrence within the 1st year after surgery.

### Surgical tools

**Figure 1:**
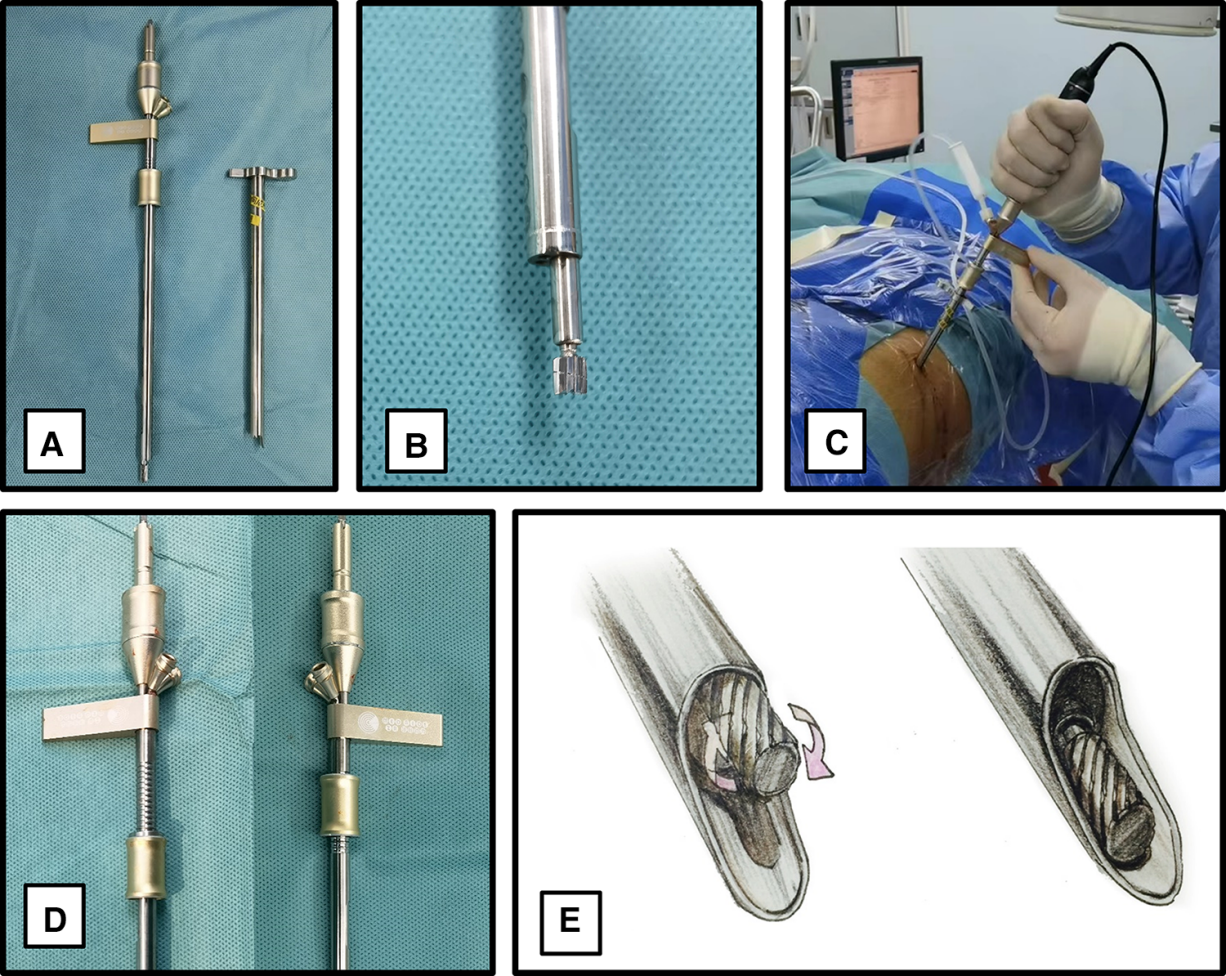
Power-aided reciprocating burr instrument. (**A**): A power-aided reciprocating burr. (**B**): An decentered cylindrical burr head. (**C**): Four main components of the instrument. (**D**): The control apparatus, which can slide on the graduated scale. (**E**): The movement mode of the decentered cylindrical burr after connecting the handle is reciprocating.

To perform foraminoplasty in a rapid, safe, and standardized manner, we used a patented specially designed instrument (Guizhou Zirui Technology Co. LTD, Gui Zhou, China) consisting of a power-aided reciprocating burr (Model: PWMXT45190Q; the diameter of the burr head is 4.5 mm, the effective length of the burr is 190 mm, the recommended speed is 30,000 r/m), a protective cannula (Model: TD75; inner diameter: 7.5 mm, outer diameter: 8.8 mm, length: 175 mm), a handle (maximum power 100 W, speed 10,000–30,000 r/min) and a flush device (Figures 1A-C). The power-aided reciprocating burr has two unique designs. One is an decentered cylindrical burr head, with which the reciprocating motion avoids soft tissue entanglement and damage (Figures 1B–E and Supplementary Video S1). The other apparatus is the control apparatus, which has a graduated scale at the tail end of the burr stem. A control apparatus perpendicular to the burr stem can slide on the graduated scale (Figure 1D). The depth adjustment range is 24 mm. The burr works inside the protective cannula, avoiding any damage to nerve roots. The JOIMAX system (JOIMAX GMBH, Karlsruhe, Germany) was used in TELD.

### Surgical procedure

The surgical method was an improvement of the TESSYS technique. Combined local anaesthesia and intravenous anaesthesia were used. The patient was placed in the lateral decubitus position with knee and hip flexion. The operating bed was folded to open the ipsilateral intervertebral foramen. The skin entry point was usually approximately 8 to 12 cm from the midline. The entry point depended on the patient's body size, location of the herniated disc, and foraminal dimension. Considering the connection lines of the articular processes' lateral perspective as safety lines by using C-arm x-ray fluoroscopy, all entry points were on the dorsal side of the connection lines of the articular process apexes to avoid damaging thoracic and abdominal organs and blood vessels.

The skin, subcutaneous tissue, and tissues surrounding the articular process were anaesthetized using 1% lidocaine. Deep fasciae and muscle tissues were anaesthetized using 0.375% ropivacaine. An 18G puncture needle was inserted in the intervertebral disc from the “safe triangle” *via* the apex of the superior articular process. After administering 10 ml of 0.5% lidocaine in the intervertebral foramen, the needle was replaced with a 1 mm guidewire. The skin at the insertion site was cut open (approximately 8–10 mm) using a scalpel, and a pencil-like guide rod was inserted into the intervertebral foramen along the guide wire. A special protective cannula matching the power-aided reciprocating burr was passed over the pencil-like guide rod and advanced with twisting motions to the intervertebral foramen. After that, the protective cannula was further rotated and advanced through the lower half of the intervertebral foramen between the SAP and posterior rim of the upper endplate of the distal vertebrae.

**Figure 2:**
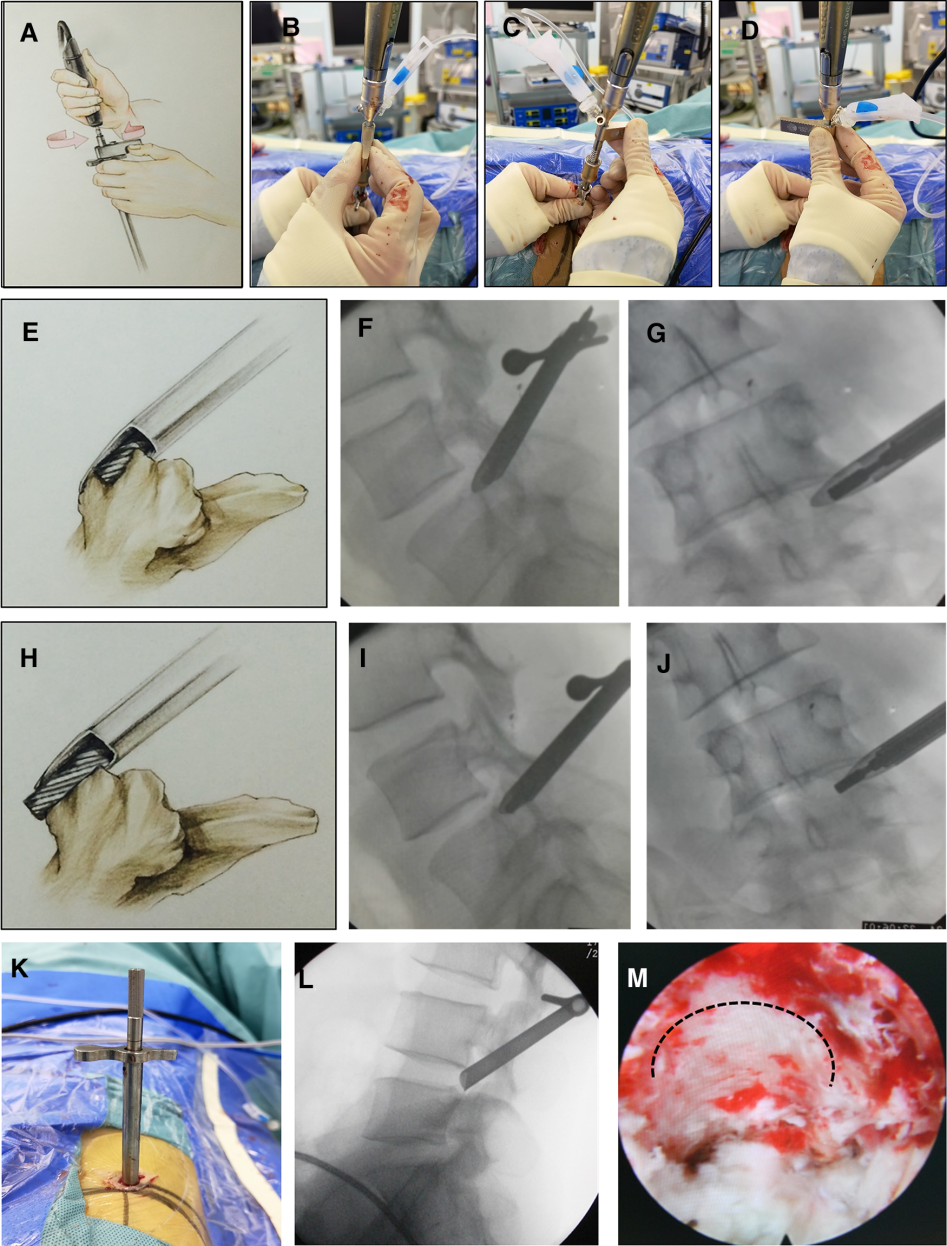
The surgical procedure of modified lumbar foraminoplasty using a power-aided reciprocating burr (L4/5). (**A-D**): Due to the design of the decentered cylindrical burr head, the burr was rotated to approximately 180° to remove the bone of the superior articular process and enlarge the intervertebral foramen. (**E-J**): The control apparatus, which has a graduated scale at the tail end of the burr stem, allows precise control of the depth of resection of the superior articular process cortex without damaging the nerve roots. (**K**): The working cannula was inserted along the pencil-like guide rod. (**L**): The tip of the working cannula should be fixed on the posterior rim of the upper endplate of the distal vertebra in the lateral fluoroscopic view. (**M**): The bone wall on the superior articular process was smooth and regular; the heat generated by the drill reduced cancellous bone bleeding.

The handle and the reciprocating burr were connected (Figure 2A). The initial depth determined by the control apparatus was set to be the appropriate size of the corresponding articular process based on preoperative CT measurements. The rotation speed was set at 30,000 rpm. The decentered burr was rotated to approximately 180° to remove the articular process bone in the channel (Figures 2B-D and Supplementary Video S2). To avoid nerve root injury, the facet joint cortex was not simultaneously penetrated (Figures 2E-G). The burr was used to continue sanding the superior articular process, 2 mm each time, until the bone at the ventral part of the superior articular process apex in the channel was removed (Figures 2H-J). Normal saline was intermittently injected into the canal for cooling during the removal process to prevent the high temperature from damaging the nerve root. After foraminoplasty, the working cannula was inserted along the pencil-like guide rod (Figure 2K), followed by a connection to the light source and lens after the canal was confirmed at the appropriate location (Figure 2L). Under a microscope, the fragmented soft tissue and residual bone pieces were removed. The bone wall on the superior articular process was smooth and regular, with a small number of bleeding spots (Figure 2M). According to the location of the herniated disc, the direction of the protective cannula can be adjusted according to the principle of targeted puncture and foraminoplasty.

After that, part of the yellow lateral ligament and herniated nucleus pulposus were removed to fully release the nerve root, followed by posterior longitudinal ligament plasty using radiofrequency ablation. Intraoperatively, patients were asked to perform a straight-leg raising test or extension test to confirm the disappearance of the symptoms before ending the operation. All patients underwent postoperative MRI one day after surgery.

### Postoperative management

The day after surgery, the patient wore a soft lumbar back brace to exercise and the postoperative MRI was re-examined. The lumbar back brace was worn for approximately 4 weeks to limit the range of lumbar motion, especially lumbar flexion and rotation, so that the ruptured annular fibrosis could achieve good healing during the rehabilitation period and recurrence of disc herniation could be decreased.

### Statistical analysis

All statistical analyses were performed using SPSS software version 21.0 (IBM Corp., Armonk, NY). Measurement data are presented as the mean ± standard deviation (SD) and were analysed by one-way ANOVA or independent samples t test. The least significant difference (LSD) test was used for pairwise comparisons. Differences were deemed statistically significant when *P* values were less than 0.05.

## Results

### Clinical outcome

All patients underwent endoscopic surgery successfully. Among the 432 patients, radicular outer membrane damage was observed in 6 cases, and 1 case had hernia of the nerve tract. Except for this patient with aggravation of postoperative numbness, the postoperative neurological symptoms of all patients were significantly improved. The mean VAS scores for low back pain and leg pain and ODI scores were significantly decreased 6 w post-operatively and were maintained until 12 months post-operatively compared to preoperative scores (*P* < 0.05) (Table 1).

**Table 1:** Changes in preoperative and postoperative ODI, VAS scores of low back pain and leg pain.

Variables	Preoperative	6 w Postoperative	6 m Postoperative	12 m Postoperative	F Value
VAS of low back pain	5.23 ± 0.64	2.67 ± 0.51^a^	2.41 ± 0.48^a^	2.23 ± 0.41^a^	3181.460*
VAS of leg pain	7.79 ± 0.51	2.53 ± 0.45^a^	1.89 ± 0.34^a^	1.18 ± 0.35^a^	22,800.602*
ODI (%)	38.04 ± 5.12	12.12 ± 1.94^a^	6.68 ± 2.59^a^	6.82 ± 2.52^a^	9009.301*

* ANOVA.

^a^ LSD test, *P* < 0.05, compared to Preoperative. w-week; m-month.

*P* < 0.05.

### Learning curve and foraminoplasty time

**Figure 3:**
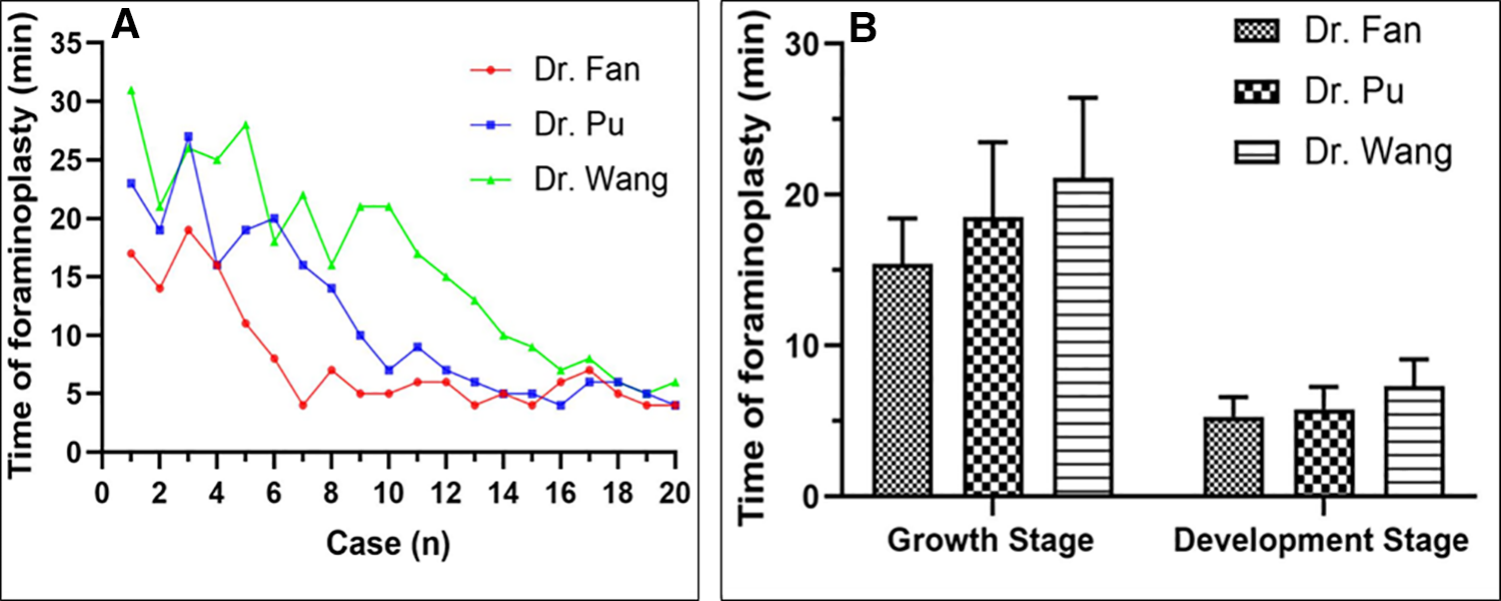
Time of foraminoplasty and number of surgical cases among three surgeons. (**A**): With the increase in the number of operations completed, the time spent by all three surgeons on intervertebral foraminoplasty decreased. (**B**): There was no significant difference in foraminoplasty time among these three surgeons during the same growing period.

**Figure 4:**
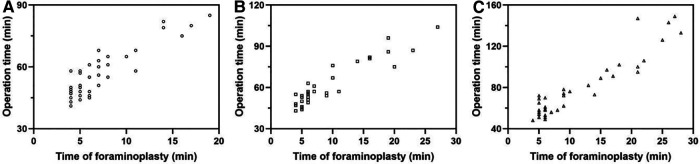
Time of foraminoplasty and operation time among three surgeons. (**A**): Dr. Fan. (**B**): Dr. Pu. (**C**): Dr. Wang.

As shown in Figure 3, with the increase in the number of operations completed, the time spent by all three surgeons on intervertebral foraminoplasty decreased (*P* < 0.05) (Figure 3A). All three doctors involved in the study had substantial experience in traditional open spinal surgery. Dr. Fan, who has 20 years of experience in TELD, needed 5 cases to move from the growth period to the development period. For Dr. Pu, with 13 years of working experience in TELD, 9 cases were needed, and 13 cases were required for Dr. Wang, who has no experience in TELD. Surprisingly, there was no significant difference in foraminoplasty time among these three surgeons during the same growing period (*P* > 0.05). For the same doctor, the foraminoplasty time of the development period was significantly shorter than that of the growth period (*P* < 0.05) (Figure 3B, Table 2, Figure 4). In addition, the time of TELD surgery for the same type of disc herniation corresponded to the time spent on foraminoplasty (Figure 4).

**Figure 5:**
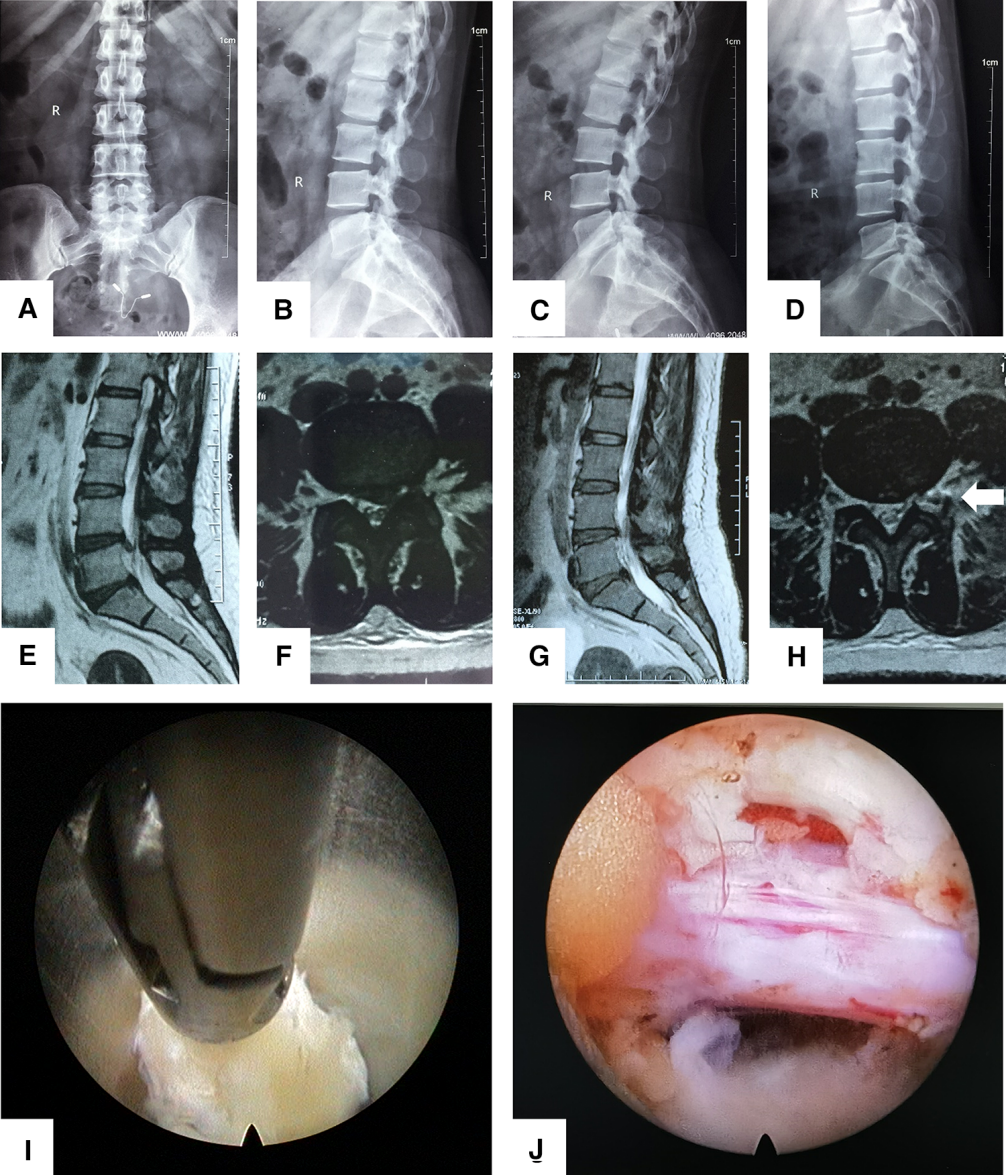
A 34-year-old female patient had radiating pain in the left lower extremity. **(A-D**): She underwent lumbar anteroposterior and lateral x-ray radiographs and lumbar overflexion-extension x-ray radiographs. The imaging data showed no lumbar instability. (**E-F**): Preoperative axial and sagittal MRI (T2WI) results showed L4/5 LDH with nerve root compression. (**G-H**): The postoperative MRI scans (1 day after surgery) of this patient show that the herniated intervertebral disc resection was satisfactory, and the structure of the lumbar facet joint was fully preserved (white arrow). (**I-J**): The herniated intervertebral disc was removed under endoscopy.

**Table 2:** Time of foraminoplasty by three surgeons during the different stages.

Surgeons	Growth stage		Development stage		*t* Value
	Patient number	Mean time of foraminoplasty	Patient number	Mean time of foraminoplasty	
Dr. Fan	5	15.4 ± 3.05	15	5.3 ± 1.29	7.170*
Dr. Pu	9	18.2 ± 4.99	11	5.8 ± 1.47	7.200*
Dr. Wang	13	21.1 ± 5.33	7	6.4 ± 1.27	9.423*
F Value		2.621^#^		1.608^#^	

* Independent-samples *t*-test, *P* < 0.05.

^#^ ANOVA, *P* > 0.05.

### Discussion

Regarding clinical outcomes, the present study showed that all patients benefited from modified TELD using a power-aided reciprocating burr as shown by the VAS scores for low back pain and leg pain and ODI scores. In addition, no severe sequelae were observed post-operatively. Compared to traditional endoscopic surgery, this modified technique showed its superiority in effectiveness and feasibility (Figure 5). For the learning process, the results showed that the number of surgical cases required for maturation was similar among surgeons with different levels of experience. For beginners, the number of surgical cases required from initiation to maturity was only thirteen. Furthermore, surgical safety with this modified technique was extremely high, even for beginners. In the present study, only one patient suffered increased numbness after surgery. In addition, once in maturity, there was less difference in the time of foraminoplasty and operation between beginners and senior surgeons. Compared to the steep learning curve of other modified endoscopic surgeries or procedures (18–20), the present modified technique of foraminoplasty was more friendly to beginners in both safety and operation time.

Hoogland et al. (9, 21) invented the TESSYS technique, which uses a graded trephine to gradually widen the foramen. Nevertheless, even with a protective working cannula, it carries the risk of injury to the exiting and traversing nerve root, which may produce leg pain and neurological dysfunction in the affected extremity. Many studies have made relevant changes to improve the safety of this method. Li et al. (14) invented a specially designed instrument for modified PLF with graded duck-mouth-like protective cannulas, which are placed on the ventral side of the SAP, excluding the exiting nerve root from the working zone of the trephine. It is important to note that although tools have improved the safety of foraminoplasty with a trephine, the lack of experience for beginners may still damage the dural sac and nerve roots (22, 23). The novel technique proposed by the present study has several potential advantages to improve the safety of foraminoplasty for beginners. First, due to the reciprocating dynamic property of the burr, there is almost no damage to the soft tissue. After the ventral bone of the superior articular process is completely removed, the risk of injury to the spinal nerve or dural sac can be effectively avoided (Supplementary Video S1). Second, the limited device depth of the burr itself can avoid the risk of the tip of the drill suddenly piercing the spinal canal and crushing the nerve root and can accurately control the thickness of the bone in the subsequent resection. Third, the new instrument is equipped with a flushing device, which can avoid the burn of soft tissue caused by high temperature while using the burr.

Advances in endoscopic equipment have improved the optical system and provided the foundation for developing other endoscopic surgical techniques (11, 12). When a burr or trephine is utilized fully endoscopically, important structures in the foramen are not damaged (24–26). Compared to trephine use under fluoroscopic guidance, fully endoscopic trephine use reduces the risk of freehand manipulation for the beginner. Because safe penetration of cortical bone requires an accumulation of surgical experience, beginners do not have good control over the depth of the trephine into the intervertebral foramen which may cause damage to nerve roots. With fully endoscopic trephine, beginners can observe the removal of the SAP under direct vision. When the bone moves concentric circles with the trephine, it indicates that the cortical bone has been penetrated, thus avoiding further inserting the trephine into the intervertebral foramen. Nevertheless, an important issue for beginners is that once bleeding occurs, it becomes difficult to continue the procedure (27). Foraminoplasty can be performed using an endoscopic drill to remove parts of the articular processes under direct vision. Choi et al. (28) employed this technique to treat 59 patients with good results. Some studies have shown that it can protect the nerve and dural sac more safely (29, 30). Nevertheless, the surgeon must be very familiar with the anatomy of the foraminal region. This ability is often lacking in beginners, which can cause beginners to lose their direction under the endoscope. Endoscopic foraminoplasty with tiny tools and burrs is a time-consuming procedure because of the restriction of the working channel of the rigid endoscope. The original aim of our research was to further improve the speed and safety of foraminoplasty so that beginners can master it quickly. From the tool design and research results, our method has the speed of trephine foraminoplasty under fluoroscopic guidance and the safety of full endoscopic foraminoplasty.

Nevertheless, we found that this method also had shortcomings in practice. Due to the limitation of the channel and the size of the burr, the amount of the superior articular process removed at one time is relatively small. For some severely prolapsed disc herniations, multiple foraminoplasties may be necessary. In addition, although the reciprocating dynamic property of the burr is very safe, there is still a risk of nerve root injury although it is rare (6/432). In the future, we aim to continuously improve the design of the burr to make it more convenient to construct the channel under an endoscope. Furthermore, as the technology becomes sufficiently mature, large-scale prospective studies will be necessary to fully assess its performance in clinical applications.

## Conclusion

In summary, current clinical data demonstrated the safety and efficacy of modified TELD using a power-aided reciprocating burr for treating LDH and showed that this technique significantly reduces the learning curve for beginners when performing foraminoplasty. This provides an alternative in clinical practice.

## Data Availability

The original contributions presented in the study are included in the article/**Supplementary Material**, further inquiries can be directed to the corresponding author/s.
